# From screen to target: insights and approaches for the development of anti-virulence compounds

**DOI:** 10.3389/fcimb.2014.00139

**Published:** 2014-09-30

**Authors:** Katherine S. H. Beckham, Andrew J. Roe

**Affiliations:** Glasgow Biomedical Research Centre, Institute of Infection, Immunity and Inflammation, College of Medical, Veterinary and Life Sciences, University of GlasgowGlasgow, UK

**Keywords:** infection, anti-virulence, secretion, inhibitor, *Escherichia coli*

## Abstract

A detailed understanding of host-pathogen interactions provides exciting opportunities to interfere with the infection process. Anti-virulence compounds aim to modulate or pacify pathogenesis by reducing expression of critical virulence determinants. In particular, prevention of attachment by inhibiting adhesion mechanisms has been the subject of intense research. Whilst it has proven relatively straightforward to develop robust screens for potential anti-virulence compounds, understanding their precise mode of action has proven much more challenging. In this review we illustrate this challenge from our own experiences working with the salicylidene acylhydrazide group of compounds. We aim to provide a useful perspective to guide researchers interested in this field and to avoid some of the obvious pitfalls.

## Introduction

The treatment of bacterial infections has become more challenging due to the increased prevalence of antibiotic-resistant strains and a stark reduction in the development of novel anti-bacterials. The current armory of compounds inhibit enzymes that are often essential to the survival of the pathogen, for example β-lactams and aminoglycosides that target bacterial cell wall biosynthesis and translation respectively. As these processes are essential for growth, the selective pressure imposed by antibiotics is strong, and the development of resistance mechanisms high. The identification of novel targets that are not essential for survival *per se* is therefore becoming an active area of research.

The AV approach is one that specifically targets “virulence factors” used by pathogens to facilitate the infection process. The application of AV compounds against factors such as quorum sensing, adhesins, and secretion systems has been tested, however the development of these compounds is still in the early stages. Whether targeting virulence factors will lead to lower selective pressure for the generation of resistance is an interesting question and has been scrutinized recently (Allen et al., 2014). Certainly anti-virulence (AV) approaches can have merit, for example when the use of traditional antibiotics is not appropriate. The clinical symptoms associated with Enterohaemorrhagic *Escherichia coli* (EHEC) infections have been shown to increase in severity following administration of certain antibiotics. This is a result of the release of Shiga-toxin following bacterial lysis (Zhang et al., 2000).

The focus of this review is the development of AV compounds that inhibit the Type Three Secretion System (T3SS), a virulence factor important for the pathogenicity of several Gram-negative pathogens, including *Salmonella* spp., *Yersinia* spp. and pathogenic *E. coli*. Here we will describe the different approaches used to identify AV compounds, along with their respective targets, and the various methods of target validation, with particular emphasis on the experience we have gained from working on a class of T3SS inhibitors, the salicylidene acylhydrazides.

## The type three secretion system

The T3SS is a key virulence determinant for a diverse range of Gram-negative pathogens. Species as distinct as *Yersinia* and *Erwinia* use the T3SS to secrete and inject pathogenicity proteins into the cytosol of eukaryotic host cell (Hueck, 1998). Whilst the core apparatus proteins of the T3SS are relatively conserved, the functions of the secreted effector proteins are highly species-specific. EHEC and enteropathogenic *E. coli* (EPEC) induce gross reorganization of the actin cytoskeleton of host-epithelial cells leading to the formation of attaching and effacing (A/E) lesions that act as “pedestals” allowing intimate attachment of the bacteria to the host. Attachment is largely achieved by the translocation of effector proteins such as Tir (the translocated initimin receptor). In both EHEC and EPEC, the entire T3SS is chromosomally encoded by a pathogenicity island called the locus of enterocyte effacement (LEE) (McDaniel et al., 1995). This T3SS is genetically quite distinct from that of *Yersinia* species, the “Ysc-Yop” system, which is plasmid encoded and regulated by different environmental signals (Lindler, 2004).

## The search for T3SS inhibitors

Deletion of the T3SS has a profound effect on the virulence potential of Gram-negative pathogens *in vivo*, making its inhibition an attractive prospect. Initial screens for a T3SS inhibitor made use of a high throughput (HTP) approach that tested large chemical libraries, consisting of both synthetic and natural compounds, against whole bacteria (Linington et al., 2002; Kauppi et al., 2003; Nordfelth et al., 2005). The use of a bacterial screening model overcomes several problems associated with drug discovery, for example cell-permeability or drug-efflux. Several of these screens employ the use of a transcriptional-reporter assay, which couples the expression of virulence genes into a fluorescent or luminescent read-out that can be easily quantified in a HTP manner.

The first reported chemical inhibitor of the T3SS was identified in 2002 by Linington et al. who screened chemical extracts from the marine sponge *Caminus sphaeroconia* against EPEC (Linington et al., 2002). The screen looked for compounds that decreased the secretion of EspB, a T3SS protein, and displayed no antibacterial activity. The product caminoside (Table 1; depicts key compounds described in this review) was found to have these properties with an IC_50_ of 5.1 μg/ml. Despite the promise of this caminoside, its cellular targets were not identified due to the difficulty of synthesizing this natural compound (Zhang et al., 2010).

**Table 1:** **Anti-virulence compounds discussed in this review**.

**Compound**	**Structure**	**Source**	**Phenotype tested**	**Effective against**	**IC_50_**	**References**
Caminoside	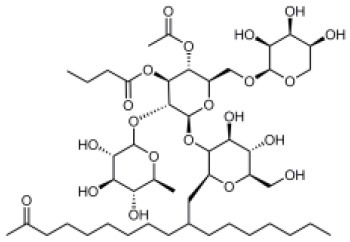	Marine sponge (*Caminus sphaeroconia*)	Effector protein secretion (EspB)	EPEC	5.1 μg ml^−1^	Linington et al., 2002
Aurodox	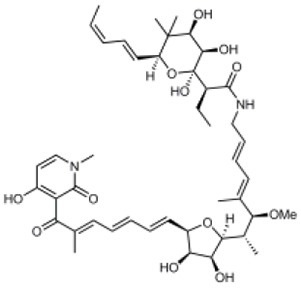	*Streptomyces* sp. extract	Effector protein secretion (EspB) T3SS- mediated haemolysis	EPEC	1.8 μM	Kimura et al., 2011
				*C. rodentium*		
Guadinomines	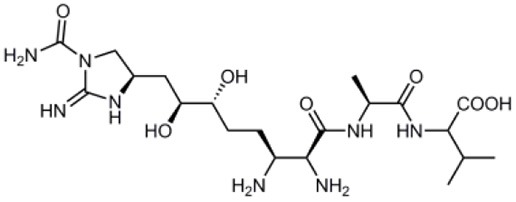	*Streptomyces* sp. extract	T3SS induced haemolysis	EPEC	<0.01 μg ml^−1^	Iwatsuki et al., 2008
INP0010 / ME0052	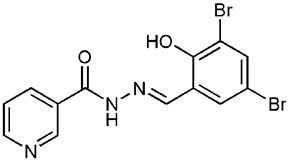	Synthetic compound library (ChemBridge)	Effector protein secretion (Yop, EspB)	*Yersinia*	25 μM	Nordfelth et al., 2005
				*Salmonella*		
				EHEC		
INP0031 / ME0055	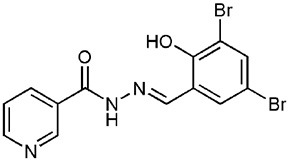	Synthetic compound library (ChemBridge)	Effector protein secretion (EspB, Tir)	EHEC	25 μM	Hudson et al., 2007
INP0341	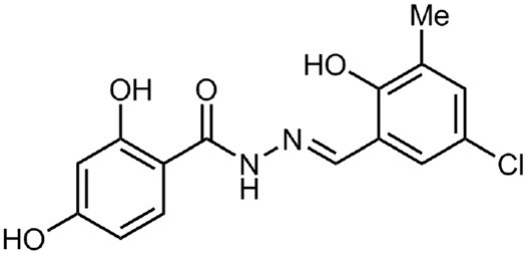	Synthetic compound library (ChemBridge)	Intracellular invasion assay	*Chlamydia*	<50 μM	Slepenkin et al., 2007
INP0400	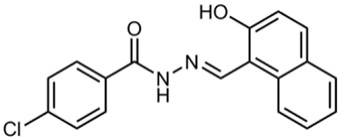	Synthetic compound library (ChemBridge)	Effector protein secretion (Yop, EspB), Intracellular invasion assay	*Yersinia*	25 μM	Muschiol et al., 2006; Negrea et al., 2007; Slepenkin et al., 2007; Veenendaal et al., 2009
				*Chlamydia*		
				*Salmonella*		
				*Shigella*		
INP0402	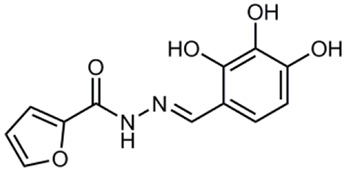	Synthetic compound library (ChemBridge)	Intracellular invasion assay	*Shigella*		Veenendaal et al., 2009
INP0403 / ME0053	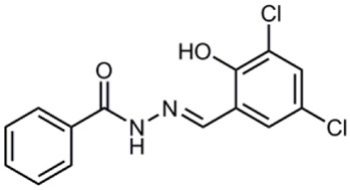	Synthetic compound library (ChemBridge)	Effector protein secretion (EspB), SpI1 expression	*Salmonella*	25 μM	Tree et al., 2009; Layton et al., 2010; Wang et al., 2011
				EHEC		
INP0406	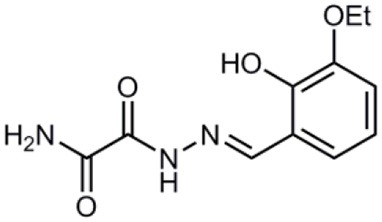	Synthetic compound library (ChemBridge)	Intracellular invasion assay	*Chlamydia*		Slepenkin et al., 2007

Several other natural products have been shown to decrease the expression of the T3SS. Aurodox, produced by *Streptomyces goldiniensis*, was recently shown to inhibit EPEC T3SS mediated hemolysis *in vitro*, with an IC_50_ of 1.8 μM (Kimura et al., 2011). Aurodox was also shown to be effective *in vivo* when tested in a mouse model of infection using the natural mouse pathogen *Citrobacter rodentium*, where mice treated with Aurodox survived a lethal bacterial load (Kimura et al., 2011). Treatment of Gram-negative species with Aurodox resulted in a specific decrease in expression of the T3SS suggesting that it may be interacting with a T3SS transcriptional regulator. Another class of compounds produced by a *Streptomyces* species (K01-0509) are the guadinomines, which were shown to inhibit T3SS in EPEC *in vitro* with IC_50_ values of lower than 0.01 μg/ml (Iwatsuki et al., 2008). Since the guadinomines appear to be highly potent with no antibacterial activity they are attractive lead compounds, however their efficacy *in vivo* has yet to be confirmed.

One of the most extensively studied group of AV compounds are the salicylidene acylhydrazides (SA), a class of inhibitors that were identified from a chemical screen of 9400 compounds carried out by Kauppi et al. at the University of Umeå (Kauppi et al., 2003). The screen was performed on *Y. pseudotuberculosis* expressing a *yopE*-luciferase transcriptional fusion, where the *yopE* promoter was fused to the *luxAB* cassette. YopE is a secreted effector protein; therefore a decrease in luciferase activity from the *yopE* promoter was correlated to reduced expression of the T3SS. This assay provided a rapid system to monitor processes regulating secretion-specific transcription. However, like all transcriptional reporters, it is rather indirect and does not provide data on whether the T3SS is functional and secreting effectors. Compounds that showed no antibacterial activity were characterized further, leaving four lead compounds from the initial screen. These were all demonstrated to decrease the secretion of effector proteins (YopE, YopD and YopH) in a dose dependent manner with an IC_50_ of less than 50 μM. Owing to the structural similarity between the Ysc T3SS apparatus and the flagellum, the compounds were tested for inhibition of motility. Only one of the four compounds, INP0010/ME0052, was shown to have an effect on motility, which was interpreted by some groups to indicate that the compounds are binding to a related structural component or to a common regulator of these systems (Kauppi et al., 2003).

Several groups later tested the SA compounds on a range of Gram-negative pathogens. The obligate intracellular pathogen *Chlamydia trachomatis* was shown to be affected by an SA compound (INP0400), identified in the original screen by Kauppi et al. (2003; Muschiol et al., 2006; Wolf et al., 2006). Treatment with this compound disrupted the normal infection cycle and prevented differentiation and multiplication in mammalian cells (Muschiol et al., 2006; Wolf et al., 2006). At the time of this study relatively little was known about the role played by the T3SS in this pathogen, thus the use of these compounds revealed insights into the importance of the T3SS in the switch between the metabolically inert “elementary body” and the infective vegetative state of the pathogen. At the time, there were only limited genetic tools available for *Chlamydia* therefore INP0400 permitted inhibition of T3SS expression throughout the developmental cycle of this pathogen (Wolf et al., 2006).

Subsequent work showed that *Salmonella enterica* serovar Typhimurium was also susceptible to the SA compounds. Dose dependent inhibition of SPI-1, one of the two T3SSs encoded in *Salmonella* (Hudson et al., 2007) was demonstrated. The compounds (e.g., INP0031/ME0055) had no effect on the growth of the pathogen *in vitro* and reduced SPI-1 mediated invasion of HeLa cells by up to 60%. The study also showed that pre-incubation of the bacteria with the compounds reduced the level of inflammation in an *in vivo* bovine intestinal ligated loop model. These data indicated that the compounds reduced the virulence of *Salmonella in vivo* (Hudson et al., 2007). Further studies in *Salmonella* by Negrea et al. confirmed the ability of the SA compound (INP0400) to inhibit SPI-1 activity (Negrea et al., 2007). They also demonstrated the compounds to be effective inhibitors of SPI-2 mediated secretion, and that treatment with the compounds reduced intracellular replication. Two of the nine compounds tested were shown to significantly reduce the motility of *Salmonella* in soft agar (Negrea et al., 2007).

Veenendaal et al. found that the SA compounds INP0402 and INP0400 were the most effective at reducing T3SS in *Shigella flexneri*, an invasive intracellular Gram-negative pathogen (Veenendaal et al., 2009). Treatment with the compound reduced its ability to invade HeLa cells and its ability to induce macrophage apoptosis, both indicative of T3SS inhibition (Veenendaal et al., 2009).

The activity of the SA compounds against EHEC was shown by our group to be effective at decreasing LEE T3S in a dose dependent manner (Tree et al., 2009). This study showed INP0031 to be the most effective compound at inhibiting LEE T3S and A/E lesion formation. All of the compounds tested (INP0010, INP0103, INP0401 and INP0031) increased the production of flagella (Tree et al., 2009). The observation that the SA compounds decreased expression of the T3SS but increased flagella expression indicated that the mechanism of action might be through a regulatory mechanism.

In summary, the SA compounds have been shown to be effective inhibitors of T3S in several species of Gram-negative pathogens. In all studies the authors observed no antibacterial activity, which is key as AV compounds should not decrease the survival of the pathogen. Several studies showed that in addition to affecting the T3SS, the expression of motility genes was also affected, however the effects between species were not consistent. Although it is known that the compounds inhibit the T3SS, the precise mechanisms underlying their mechanism of action (MOA) is yet to be elucidated.

## Proposed mechanism of action of the SA compounds

There are three main schools of thought about how the SA compounds function. Firstly, by disrupting cellular iron stores. Secondly, by directly interacting with a component of the T3SS apparatus, and thirdly, by causing dis-regulation of T3SS expression. The finding that the activity of the compounds could be reversed following the addition of iron to the cell culture media was first reported by Slepenkin et al. (2007). This study showed that the addition of iron to HeLa cells infected with *C. trachomatis* reversed the effects of the inhibitors. This effect was not seen when other divalent metal ions were added. However, these results were somewhat inconclusive since INP compounds that did not affect the T3SS in *Chlamydia* (INP0406) chelated iron to the same extent as INP0341, a potent inhibitor (Slepenkin et al., 2007). Indeed, the most promising clinical application of the SA compounds is for protection against *Chlamydia*. For this strict intracellular pathogen the SA compounds affect not only the T3SS but also growth and replication of the bacteria, almost invariably through iron sequestration (Ur-Rehman et al., 2012). When used as a vaginal biocide, SA compounds were able to significantly protect mice from a vaginal infection of *C. trachomatis* (Slepenkin et al., 2011). A similar study by Layton et al. indicated that the effect of the SA compounds could be partially reversed by the addition of iron (Layton et al., 2010). Transcriptomic analysis of *Salmonella* treated with INP0403 showed a significant increase in several genes involved in iron regulation (Layton et al., 2010). However, the addition of iron did not fully reverse the anti-SPI1 T3SS activity of INP0403. Microarray studies carried out on EHEC grown in the presence of iron found that SA compounds (INP0010 and INP0031) lead to a significant decrease in the expression of the LEE (Tree et al., 2009), thus indicating that in this case iron is not inhibiting the action of the SA compounds. Therefore, it remains unclear how iron affects the activity of the SA compounds and further work is required to clarify the effects of iron on the T3SS.

Veenendaal et al. proposed that the compounds were acting directly on a component of the T3SS. The reports of motility also being affected by the compounds led to the conclusion that the component being targeted may be one that is homologous between the T3SS and flagellar systems (Veenendaal et al., 2009). The evidence for this proposed mechanism was that following SA compound treatment of *Shigella*, the needle filaments of the T3SS were significantly shorter than for untreated cells indicating that the compounds were affecting needle assembly (Veenendaal et al., 2009). A further study by the same group sought to determine this common component in *Salmonella*. By using strains deficient in three soluble components of the flagella apparatus they aimed to identify which of these were responsible for the change in motility seen following compound treatment (Martinez-Argudo et al., 2013). However, this study was unable to show that the SA compounds directly affected flagellar components. The authors concluded that the SA compounds were not directly inhibiting T3SS or flagellar components and were most likely interacting with other targets within the cell and indirectly affecting the expression of these virulence factors (Martinez-Argudo et al., 2013).

Transcriptomic profiling of EHEC treated with 20 μM ME0052 or ME0055 resulted in the decreased expression of the five operons that comprise the LEE as well as an increase in the expression of flagellar associated genes (Tree et al., 2009). These data provided important clues as to how the SA compounds might be working. Firstly, the reduction in transcription of the entire LEE suggested that the compounds either affected the master regulator of the system (Ler) or an upstream regulator that affected Ler itself. Indirect support for this hypothesis comes from the observation that deletion mutants for LEE genes encoding proteins of the secretion system itself do not result in regulatory feedback and a reduction in LEE transcription (Deng et al., 2004). This suggests that the SA compounds are unlikely to simply bind to basal apparatus proteins, as this would not be consistent with the transcriptional changes observed. Further evidence to explain the MOA of the SA compounds comes from work investigating the type four secretion system (T4SS) of *Brucella*. T4SS have a completely different protein structure compared to the T3SS but are also important virulence factors for many Gram-negative pathogens (Baron, 2006). Based on the knowledge that dimerization of the assembly factor VirB8 is a prerequisite for VirB8 function, a bacterial two-hybrid assay was established (Smith et al., 2012). This allowed several inhibitors of VirB8 dimerization to be identified including B8I-2, a salicyidene acylhydrazide. Co-crystals of B8I-2 and VirB8 were obtained, allowing the residues critical for the inhibitory activity to be mapped. Interestingly, when seven SAs that inhibited the T3SS were tested, all were found to be inactive in the VirB8 interaction assay (Smith et al., 2012). This raises the possibility that either the SA compounds are rather promiscuous, and bind numerous proteins, or that despite the absence of obvious protein sequence similarities, both of the T3 and T4 secretion systems may be inhibited by a similar mechanism.

In summary, it is easy to assume that because one has established a screen for compounds that affect the expression of the T3SS, the compounds are directly targeting the secretion system itself. This assumption is dangerous and it is wise to determine the global effects of any compound using either transcriptomic or proteomic approaches before focusing on a subset of targets.

## Defining the mechanism of action for AV compounds

From the outline above, it is clear that whilst it is relatively easy to develop a robust screen and identify novel lead compounds, elucidating their specific mode of action is much more problematic. The first step toward unraveling the MOA of a novel compound is to identify its cellular target or targets. There are several different approaches that can be taken to investigate targets such as genetic or biochemical screening and affinity chromatography. However, when used in isolation these approaches will not always give a clear answer and in our experience we have found that using a combination of these approaches is most beneficial.

## Strengths and limitations of approaches to target identification

Most groups performing screening projects have a background in bacteriology and therefore adopt classical genetics approaches to try and understand MOA. For traditional antibiotics, simple screens are often employed to identify mutants that are resistant to the effects of the agent. Exposure of a large bacterial population to a high dose of the antibiotic will often yield “escape mutants” that are resistant to the compound (Bergstrom and Feldgarden, 2008). Alternatively, saturated transposon mutagenesis can be employed in which a bank of mutants is created and then screened for escape mutants. Genome sequencing is now routine and affordable such that mutants can be readily analyzed and the mutation identified. Ideally, the transposon would be in a gene encoding the target, directly revealing the likely MOA. However, resistant mutants can be more obscure, for example in a porin or membrane transporter that results in a lower intracellular concentration of the antibiotic (Fernández and Hancock, 2012). For AV compounds, an inherent limitation is often that the screening of mutants is far more time-consuming than for bactericidal antibiotics. By their very design, AV agents do not affect bacterial growth or survival so simply “plating out” a large population on a high concentration is not likely to produce resistant mutants that will inform the MOA.

We have utilized two different approaches to identify escape mutants. Firstly, generation of a transposon insertion library in which each mutant is screened for expression of the T3SS and secondly by screening “wild-type” isolates with a view to finding variants that are less sensitive to the AV compound. In the former, the process has proved to be time-consuming and is absolutely dependent on the quality of the screen, in our case a GFP transcriptional-reporter assay that can be run in 96-well plate format. Given that the EHEC genome carries approximately 5500 genes, there is no overcoming the large volume of work involved in screening thousands of mutants. Moreover, for each mutant that exhibits insensitivity to an AV compound, further screens must be undertaken to verify that growth is unaffected. One inherent limitation of such a screen is that, if the protein target were directly involved in the T3SS itself (such as a structural protein), then insertion of a transposon into corresponding gene would inhibit T3SS function entirely. Overall, the simplicity of the approach needs to be balanced against the time needed to be invested and should only be adopted if the screen is extremely robust.

Our second approach has been to screen a bank of clinical isolates. Selection of 18 clinical *E. coli* O157 isolates with a diverse range of phage types revealed a strain that was completely insensitive to the SA compounds. Next generation sequencing provided rapid and accurate identification of genetic differences when compared to that of reference strains. The success of this approach is largely dependent on having access to a diverse strain collection, as clonal isolates are likely to display little phenotypic variation. However, greater diversity results in more genetic differences. In our insensitive mutant there were over 1300 single nucleotide polymorphisms (Wang et al., 2011). These data were a useful comparator to other target identification approaches but, in isolation, would not be sufficient to inform of a specific target with confidence.

As discussed above, transcriptomic profiling, historically using microarrays and more recently RNA-seq, is a powerful and unbiased method to reveal insights into the global effects of any compound. Although transcriptomic profiling reveals no direct data as to the likely target protein or underlying mechanism it does provide valuable data. In particular, it provides a clear indication as to the specificity of the compound: does it affect a single operon, a network of genes or a substantial proportion of the genome? As already described, for the SA compounds it was clear that several operons, not just the LEE, were affected. These data suggested that numerous target proteins were bound or that the compounds interfered with core aspects of bacterial physiology.

Alternatives to transcriptomic studies are the well-established methods of proteomics and the relatively recent addition, metabolomics. Using two-dimensional difference gel electrophoresis (2D-DIGE) provides a sensitive and robust approach to detecting changes in protein expression (Kondo and Hirohashi, 2006). The most clear advantage being that it is possible to detect possible post-transcriptional effects caused by any compound. The major limitation is that only a proportion of the proteome can be resolved on a single gel.

Metabolomics is widely used in the pharmaceutical industry to test the effects of drugs on host-cell processes. For example, metabolomic studies have demonstrated that D-cycloserine, a second-line treatment for *Mycobacterium tuberculosis*, is rather non-specific and causes inhibition of numerous enzymes (Halouska et al., 2007), a result that might explain some of the less-desirable side effects. However, metabolomics has barely been applied to AV development. Targeted metabolomics follows changes to a specific metabolite based on some prior information, so it is unlikely to be used at an early stage or to reveal information regarding a possible MOA. However, untargeted metabolomics is discovery based and aims to monitor the entirety of the metabolome in order to identify the affected metabolites and pathways. It is feasible that metabolomics might reveal discrete changes in pathways providing insights into the global effects of an AV compound but it is likely to be employed at a later stage to investigate potential toxicity issues when MOA has been established.

**Figure 1:**
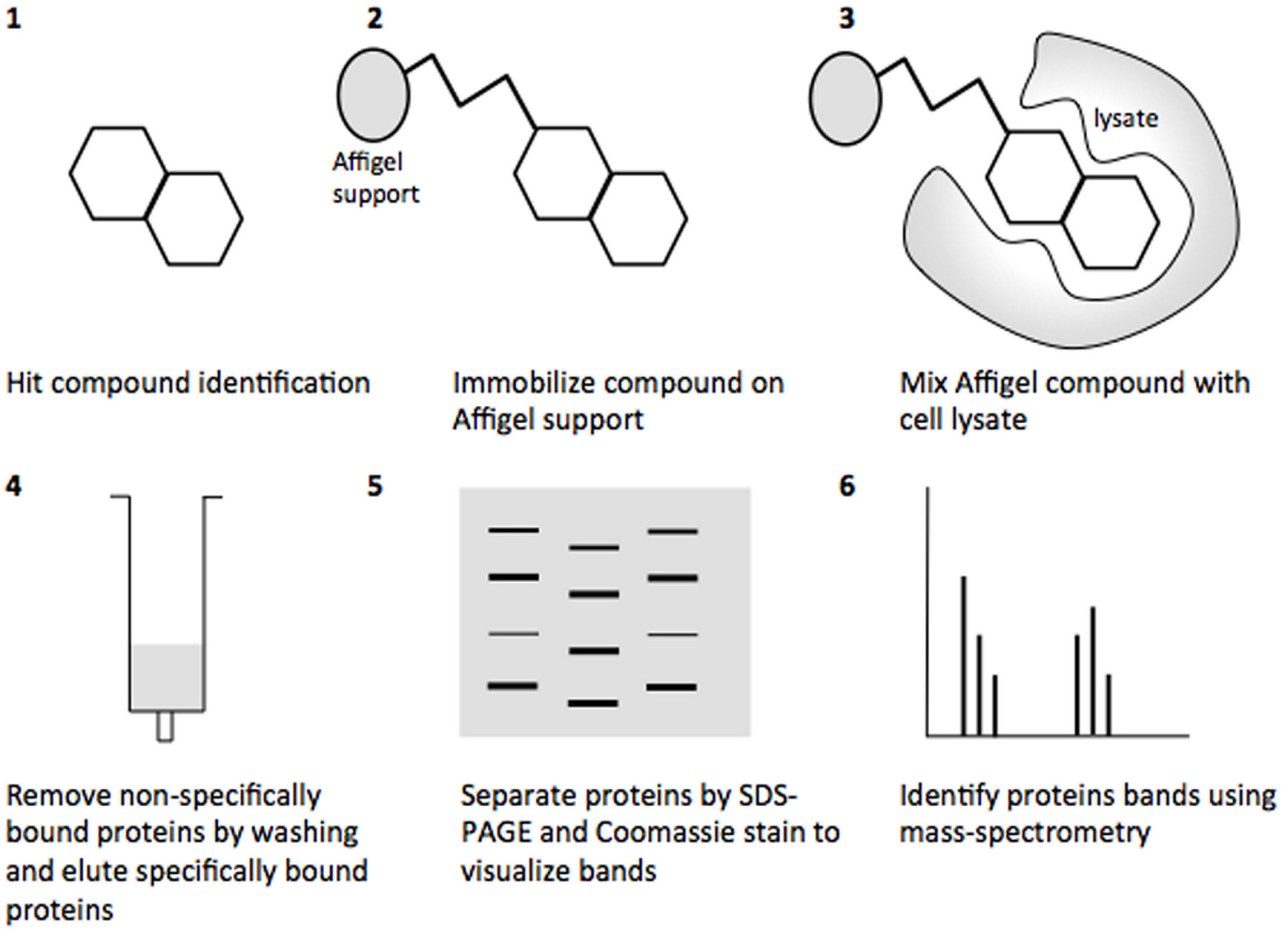
**Overview of the affinity pull-down assay to determine the binding proteins of the salicylidene acylhydrazides**. **(1)** The hit compound was identified from reporter assays screening for decreased T3SS expression. **(2)** The hit compound was attached to an Affigel support. **(3)** The Affigel labeled compound was mixed with cell lysate, allowing the putative targets to bind. **(4)** Non-specifically bound proteins were removed by washing and specifically bound proteins were eluted with high concentrations of the free compound. **(5)** Eluted proteins were resolved by SDS-PAGE and protein bands visualized by Colloidal Blue Stain and excised. **(6)** Protein bands were identified by mass-spectrometry.

Affinity chromatography is a powerful technique that enables identification of compound binding partners from whole-cell lysates. An overview of the basic steps is provided in Figure 1. A successful pull-down first requires that the compound can be attached to an immobile matrix without disrupting the activity of the compound. This is not without complications as not all of the chemical groups on the compound may be suitable for attaching a linker to immobilize the compound. Therefore, it is often valuable to conduct a structure-activity relationship (SAR) where different chemical variants are screened against whole cells in order to identify the regions of the molecule associated with the desired phenotype. In the case of the SA compounds it was found that the active groups of the compounds were located on the right hand phenol group (Table 1) (Wang et al., 2011). This knowledge allowed the design and synthesis of ME0055-Aff, an Affigel labeled derivate of the SA inhibitor. Using the Affigel labeled derivative an affinity pull-down assay of *E. coli* O157:H7 cell lystates identified 19 putative protein binding targets (Wang et al., 2011). A combination of phenotypic analyses and biophysical studies on purified proteins were used to critically assess the contribution of these putative targets to the phenotype associated with SA addition.

## Target validation

The identification of putative target proteins is a great step forward in understanding the MOA of a compound. However, it is important to confirm any interaction and, more importantly, that the target is associated with the expected phenotype. Our affinity chromatography experiments revealed multiple targets for the SA compounds (Wang et al., 2011). These were likely a mix of genuine targets, false positives but also proteins bound by the compound that did not contribute to the overall phenotype. To test this, we employed a variety of different biophysical methods. These included chemical shift nuclear magnetic resonance (NMR), X-ray crystallography, and analytical ultracentrifugation. Further approaches including surface plasmon resonance (SPR) and isothermal titration calorimetry (ITC) would also be applicable but the low solubility of the SA compounds in biologically relevant solutions made these problematic. Chemical shift NMR allowed us to demonstrate that one target protein, a thiol peroxidase called Tpx was indeed a target of the SA compounds. A discrete binding site at the dimer interface was mapped and input from collaborators helped build a model of the SA compounds bound to the oxidized form of Tpx (Gabrielsen et al., 2012), validating the affinity chromatography result. The finding that the SA compound bound at the dimer interface is consistent with the aforementioned work in *Brucella*, which showed a different SA compound inhibited dimerization of VirB8 (Smith et al., 2012). However, deletion of the gene encoding Tpx did not cause a dramatic effect on the expression of the T3SS. Some regulatory changes were seen, but not the stark reduction in expression one might expect if the target protein was central to the phenotype. Our conclusion from this work was that inhibition of T3SS activity is due to a poly-pharmacological effect on proteins involved in metabolism and that there was no single clear target that we could attribute to the phenotype. This highlights the importance of generating deletion mutants for all putative targets at the earliest opportunity. Indeed, following this approach we systematically deleted more of the genes encoding putative target proteins including AdhE, a bi-functional acetaldehyde-CoA dehydrogenase and alcohol dehydrogenase involved in central metabolism. Deletion of the gene encoding AdhE in EHEC caused a marked reduction in T3S and an elevation of flagella production (Beckham et al., 2014), both of which are phenotypes seen when the SA compounds are added to EHEC. However, there were some clear regulatory disparities when comparing the deletion of the gene encoding AdhE and when the SA compounds are added. Specifically, deletion of AdhE caused a post-transcriptional regulation of the LEE, whereas addition of the SA compound showed transcriptional repression. This difference might be attributed to a number of factors. The generation of the defined deletion results in no AdhE protein being produced. In comparison, if the SA compounds affect AdhE activity, they are unlikely to completely block both enzymatic functions. The prediction is that the metabolic flux through the pathways associated with AdhE will be different in the two cases, the deletion compared with enzymatic inhibition. However, by systematically analyzing each putative target of the SA compounds, we have found a metabolic enzyme that is clearly linked to virulence gene expression. Our working model, in agreement with other studies (Martinez-Argudo et al., 2013) suggests that the SA compounds bind several bacterial proteins and affect virulence by disrupting several core metabolic proteins.

## Conclusions

The urgent need for need anti-infective agents is one of the most pressing challenges facing the scientific community. AV agents provide one route to new classes of drugs that are targeted to specific pathogens. The availability of small compound and natural product libraries makes screening for leads relatively simple but the largest challenge remains elucidating the precise MOA. An integrated approach using both classical genetics and biochemical methodologies is most likely to reveal this valuable information and allow researchers to make the jump toward structure based drug design and ultimately, clinically-relevant drugs.

### Conflict of interest statement

The authors declare that the research was conducted in the absence of any commercial or financial relationships that could be construed as a potential conflict of interest.
